# Carbon Dots as Multifunctional Nanofillers in Sustainable Food Packaging: A Comprehensive Review

**DOI:** 10.3390/foods14173082

**Published:** 2025-09-02

**Authors:** Yuqing Wu, Wenlong Li, Yuerong Feng, Jiyong Shi

**Affiliations:** Agricultural Product Processing and Storage Lab, School of Food and Biological Engineering, Jiangsu University, Zhenjiang 212013, China; 2112318154@stmail.ujs.edu.cn (Y.W.); 18896656873@163.com (W.L.); 18342628186@163.com (Y.F.)

**Keywords:** carbon dots, smart packaging, active packaging, nanofiller, antimicrobial, antioxidant

## Abstract

Food packaging systems play a critical role in reducing resource wastage, extending shelf-life, and enhancing supply chain sustainability. Carbon dots (CDs) have emerged as promising nanofillers for sustainable active and smart packaging due to their exceptional optical properties, biocompatibility, and antimicrobial activity. This review synthesizes recent advances in CD-based food packaging technologies, focusing on their multifunctional applications and performance enhancements. We systematically analyze how CDs improve packaging materials’ mechanical strength, gas barrier properties, and functional performance (antioxidant, antimicrobial, and smart sensing capabilities). Current research demonstrates CDs’ ability to enable intelligent functions such as pH responsiveness and freshness monitoring while maintaining excellent biocompatibility. However, challenges remain in scaling up production, long-term toxicological evaluation, and matrix compatibility. Future research directions should address these limitations while exploring the full potential of CD-based multifunctional films as sustainable alternatives for next-generation food packaging systems.

## 1. Introduction

Food packaging is crucial in safeguarding food integrity and safety by preventing potential chemical, physical, and biological hazards [1]. Synthetic polymers, owing to their superior self-efficacy and ease of use, effectively fulfill the essential requirements for food protection, sealing, and containment, thus ensuring the safety and quality of food during storage and transportation [2]. Developing packaging films and coatings from biopolymers is significant for addressing the environmental and health issues associated with plastic materials [3]. However, biopolymer-based materials generally exhibit inferior barrier properties compared to plastic films [4]. In response to these challenges, researchers and technological experts in the food industry are increasingly investigating novel strategies that focus on nanotechnology-based solutions [5]. One prevalent method for overcoming these limitations involves incorporating inorganic or organic nanoparticles fillers into biopolymers.

Carbon-based nanoparticles are the most promising alternative biocompatible materials [6,7]. Carbon dots (CDs) have emerged as particularly promising carbon-based nanomaterials for food packaging applications, demonstrating superior biocompatibility and safety profiles compared to conventional metal/metal oxide nanoparticles [8,9]. CDs are mainly composed of the elements carbon, hydrogen, and oxygen, which means that they do not produce the biotoxicity associated with metal/metal oxide containing nanoparticles [10]. CDs are more suitable for food packaging applications than other nanomaterials due to their chemical inertness, higher biocompatibility, and negligible cytotoxicity [11]. In terms of metabolism, CDs do not accumulate in the body for long periods of time, thus reducing the risk of toxicity. In addition, unlike polymeric nanomaterials, CDs do not produce potential toxins during degradation. These properties are attributed to precursor materials such as chemicals (i.e., ethanol, citrate, glucosamine, and ascorbic acid) or biomass (i.e., garlic, banana, and strawberry juice) [12]. However, there are two major gaps in current research.

CDs have strong SP^2^ C-C bonding and geometrical arrangement, which improves the properties of polymer matrix when added to polymer materials in the preparation of food packaging composites [13,14]. Compared to gold nanoparticles and carbon nanotubes, the high solubility of CDs in water allows them to be uniformly soluble and dispersed in the film-forming matrix. In contrast to other carbon-based nanomaterials, CDs can retain the reactive groups of the raw material during the synthesis process, giving CDs a powerful surface functionality [13]. By synthesizing CDs, these materials not only retain their original properties, but also gain the advantages of nanomaterials. These different groups allow CDs to bind to a variety of molecules, thereby improving their functionality [15]. For example, the hydrophilic bonds formed between CDs and the polymer matrix significantly improved the bioactivity and physical barrier properties of the composite membranes [16]. In addition, the redox-active surface of CDs provides antioxidant capacity through electron transfer, while charged functional groups and nanoscale dimensions confer antimicrobial activity. Comparative studies suggest that CDs maintain the functional benefits of traditional NPs while overcoming their key limitations in food contact applications, though further research is needed to optimize their performance–cost ratio for industrial-scale adoption.

**Figure 1:**
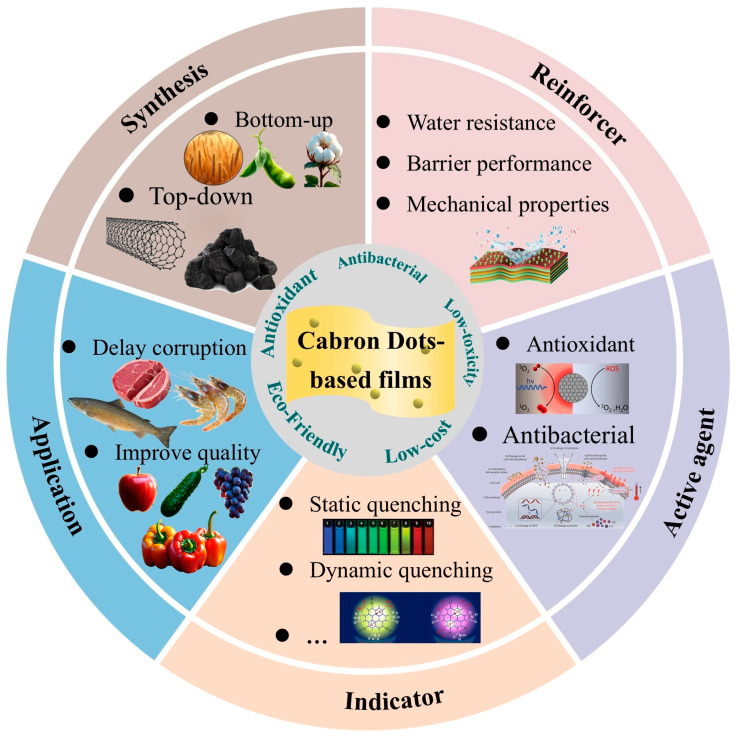
Schematic illustration of CD-based film for food packaging application.

This study systematically reviews the latest research progress of CDs’ nanomaterials in food packaging, focusing on their multifunctional properties in packaging films, including mechanical properties, barrier capacity, antimicrobial, and antioxidant functions. The study analyzes in detail the innovative applications of CDs as active ingredients and smart indicators in food packaging, and provides an in-depth analysis of their practical application cases in active packaging and smart packaging systems. In particular, this study also systematically evaluates the biosafety issues of CD materials in packaging applications and proposes corresponding risk control strategies. Previous studies have mainly focused on the synthesis process of CD materials and their basic packaging applications. There is a lack of detailed elaboration on the use of CDs as reinforcing agents to improve the physical properties of packaging materials and the use of CDs as indicators to monitor the freshness of food. The aim of this paper is to present recent insights into the characterization and application of CD-based nanomaterials in enhanced, active, and smart food packaging systems. A complete understanding of the mechanism of action of CD fillers in bio-based polymer composites can be obtained through this study. This study provides a theoretical basis for the development of novel functional food packaging materials (Figure 1).

## 2. Preparation of CDs for Food Packaging Applications

### 2.1. Top–Down Approaches

The top–down method for the synthesis of CDs is a strategy for cutting large-size carbon materials (e.g., graphite, carbon nanotubes, activated carbon, etc.) into nanoscale CDs by physical or chemical methods, which mainly include arc discharge, laser ablation, electrochemical oxidation, and chemical oxidation [17]. Taking the arc discharge method as an example, the graphite electrode is evaporated by a high-voltage arc in an inert atmosphere, and the resulting carbon fume is oxidized by acid and surface passivation to obtain fluorescent CDs with a size of 2–10 nm, but the product usually requires further purification to remove by-products. The laser ablation method uses the pulsed laser bombardment of graphite targets to generate CDs in water or ammonia and enhance their dispersion and fluorescence properties through surface modification. The electrochemical method oxidizes graphite electrodes by applying a voltage of 3–5 V and strips the carbon dots in the electrolyte NaOH or ethanol, with good monodispersity of the product. The chemical oxidation method uses a strong acid or an oxidizing agent to oxidatively cut the carbon source, and then obtains CDs by alkali neutralization and dialysis purification [18]. While top–down approaches offer advantages in precursor availability and scalability, their technical and economic limitations warrant careful consideration. From a scalability perspective, these methods demonstrate excellent production capacity and relatively low material costs (USD 50–200/g) due to the use of commodity carbon sources like graphite. However, the apparent cost advantage of top–down synthesis is partially offset by significant purification costs (30–40% of total production) and notable environmental concerns. Chemical oxidation methods generate substantial acidic waste containing residual oxidants (e.g., HNO_3_, H_2_SO_4_), requiring specialized neutralization and disposal protocols. Electrochemical approaches reduce chemical waste but increase energy consumption (15–20 kWh/g), particularly when pursuing narrow size distributions. In addition, studies have shown that CDs prepared using a top–down method may contain trace amounts of metal residues. Even after purification, their cytotoxicity (cell survival rate of 70–85%) is significantly higher than that of CDs prepared using a bottom–up method (>90%) [19].

### 2.2. Bottom–Up Approaches

The bottom–up synthesis of CDs is a method of building nanostructures by the carbonation of molecular precursors, which is usually divided into various routes such as solvothermal, microwave, pyrolysis and template methods. In the solvothermal method, for example, small-molecule organic precursors such as citric acid and urea are first dissolved in water or an organic solvent [20]. The mixed solution is then placed in a high-pressure reactor and heated at 120–250 °C for several hours. During this process, the precursors form CDs through dehydration, polymerization, and carbonation reactions, and their surfaces are enriched with carboxyl groups, amino groups, and other functional groups. At the end of the reaction, carbon dots with a uniform particle size (2–10 nm) and fluorescent properties are obtained by dialysis or centrifugal purification. The microwave method uses microwave radiation to accelerate the cross-linking and carbonization of precursor molecules, and the reaction time can be reduced to minutes [21]. The pyrolysis method involves treating the solid precursor at high temperature (300–400 °C) in an inert atmosphere and then oxidatively stripping it to obtain CDs, while the template method (e.g., using mesoporous silica as a hard template) can precisely regulate the size and pore structure of CDs. These methods optimize the fluorescence quantum yield of CDs and their performance in bioimaging and sensing applications by modulating the reaction temperature, time, precursor ratio, and surface passivator. The advantage of the bottom–up approach is that the chemical structure and optical properties of the CDs can be flexibly regulated by molecular design. The bottom–up method typically uses organic small molecule precursors, avoiding the toxicity risks introduced by graphite electrodes or metal catalysts in the top–down method. 

#### Methods of Synthesis of Carbon Dots from Food-Derived Materials

Bio-based CDs have attracted considerable attention in food packaging applications due to their distinctive advantages. These carbon-based nanomaterials are typically synthesized from natural waste materials, including fruit peels, nut shells, and dairy by-products, through eco-friendly preparation methods such as hydrothermal treatment [22]. The bio-based CDs exhibit three prominent advantages that make them particularly suitable for food packaging applications. Their abundant and low-cost raw materials enable high-value utilization of food waste streams. Green synthesis approaches eliminate the need for toxic reagents commonly employed in conventional chemical synthesis. Most importantly, they demonstrate exceptional biocompatibility and biodegradability, significantly mitigating potential safety concerns associated with packaging materials. Table 1 summarizes the currently reported bio-based CDs with their characteristic properties and potential applications. Table 2 lists the synthesis of CDs using various methods with multiple bio-based materials.

## 3. Strategies for the Preparation of CD Composite Films

CD composite films can be prepared by a variety of methods, including solution casting, spin-coating, layer-by-layer self-assembly, in situ synthesis, and vacuum filtration [27,36]. Solution casting involves uniformly dispersing CDs in a polymer solution, mixing with ultrasonic waves or stirring, and then pouring into a mold where the solvent evaporates at room temperature or under heated conditions to form a film. However, the drying speed must be precisely controlled to prevent cracking. Solution casting is the most commercially viable method, producing mechanically robust films that are ideal for packaging fresh produce. The solution-cast CDs/polyvinyl alcohol film enhances its mechanical properties, barrier properties, and thermal stability, and endows the composite film with antioxidant and antibacterial properties, thereby extending the shelf life of banana, jujube, and fried meatballs [37]. The spin-coating method uses high-speed rotation at 2000 to 4000 rpm to spread the CD–polymer mixture uniformly on substrates such as silicon wafers or glass, and is particularly suitable for the preparation of ultrathin, controllable films with thicknesses of tens to hundreds of nanometers. Spin coating can produce ultra-thin (*n* < 100 nm) and highly uniform films, making it ideal for smart indicators. Zhang et al. used casting and electrospinning methods to prepare two types of highly hydrophobic double-layer indicators. The nanofiber films prepared by electrospinning exhibited higher UV blocking ability (ΔE < 4), faster decay response, and higher safety (cell viability > 80%) [38]. The layer-by-layer self-assembly method is based on the principle of electrostatic interactions, whereby the thickness and composition of the film can be precisely regulated by alternately depositing oppositely charged CDs and polyelectrolytes. Layer-by-layer self-assembly excels in creating gas-selective barriers for modified atmosphere packaging, while in situ synthesis yields films with superior interfacial bonding strength (peel resistance >3.5 N/mm) for flexible packaging applications. The in situ synthesis method blends a carbon source, such as citric acid, with a polymer precursor to simultaneously generate CDs and form composite films with strong interfacial bonding during the heat treatment process. For high loading requirements, the vacuum filtration method filters the CD dispersion through negative pressure to form dense films on porous substrates such as cellulose acetate membranes. To further enhance the film properties, cross-linking agents such as glutaraldehyde or UV irradiation treatments can be used to enhance the mechanical strength and stability. Crosslinking strategies (e.g., 0.5–1.5% glutaraldehyde) can further enhance wet strength by 60–75%, although residual crosslinker levels must remain below 50 ppm to meet EU 10/2011 regulations [39]. These preparation strategies can be tailored to optimize the optical, electrical, and mechanical properties of the films for different applications such as fluorescent sensing, anti-counterfeit packaging, or flexible electronics.

## 4. CD Films for Food Packaging Applications

Several of the aforementioned techniques for preparing CD film packaging combine the advantages of carbon nanomaterials and polymer matrices, resulting in the development of composites with a variety of complementary properties, including tunable optical properties, environmental stability, mechanical flexibility, and functional surface properties [40,41]. Based on this, CD film packaging has been widely used in biomedical labelling, environmental pollutant detection and photocatalytic preservation. This section focuses on the innovative application of CD materials in the field of food packaging, especially the performance modulation of films, the function of packaging atmosphere regulation, and the real-time visualization and detection technology of food spoilage process [42]. In addition, the mechanical and barrier properties of packaging materials could be significantly enhanced by embedding CDs into biodegradable polymer matrices for the preparation of composite films. Thanks to their fluorescence sensitivity and low biotoxicity, CD films are ideal for use as food freshness indicators, with optical signals that efficiently respond to interactions with environmental substances. CD films are expected to be a green alternative to traditional petroleum-based packaging, provided that food safety standards are met.

### 4.1. CDs as Reinforcing Agents for Modified Packaging Films

**Figure 2:**
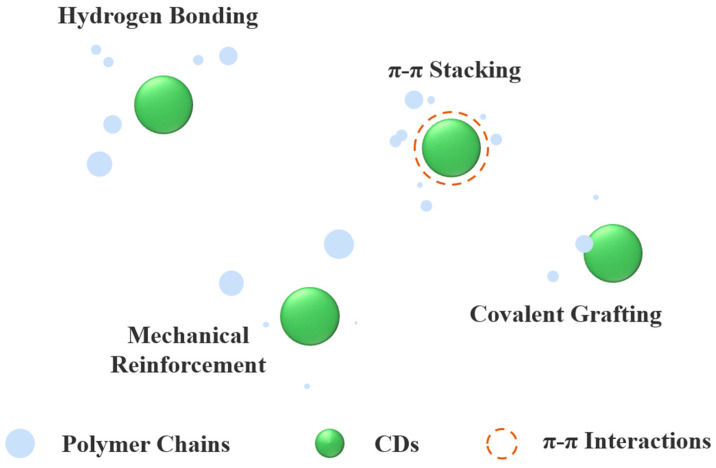
Molecular interaction between CDs and polymer matrix.

CDs enhance polymer composite performance through multifunctional mechanisms. As nanofillers, they restrict polymer chain mobility, reinforcing mechanical strength, while their ability to create tortuous diffusion pathways impedes gas and molecular permeation, improving barrier properties by sealing matrix micropores. Furthermore, interfacial interactions between CDs’ surface functional groups (-COOH, -OH, -NH_2_) and polymer chains strengthen bonding, and their rigid core structure facilitates efficient stress transfer, ensuring uniform mechanical load distribution. These synergistic effects collectively enhance the composite’s mechanical integrity and barrier performance (Figure 2).

CD-based films demonstrate unique advantages over metal nanoparticles, carbon nanotubes (CNTs), graphene, and cellulose nanocrystals (CNCs) by combining exceptional optical properties, biocompatibility, and multifunctionality in smart packaging applications. Table 3 presents a comparative analysis of CDs versus other nanoparticle fillers in composite film applications. While metal nanoparticles (e.g., Ag, Au) exhibit strong plasmonic and antimicrobial effects, they lack intrinsic sensing capabilities and raise concerns regarding metal ion migration and toxicity. CNTs and graphene offer superior mechanical strength and electrical conductivity, but their poor dispersibility, potential cytotoxicity, and high production costs limit their practicality in food-contact applications. CNCs, though biodegradable and renewable, primarily serve as structural reinforcements with limited optical or antimicrobial functionality. In contrast, CDs uniquely integrate pH-responsive fluorescence, UV-blocking properties, and mild antimicrobial activity in a low-toxicity, solution-processable format, enabling dual-mode sensing and preservation. However, CDs exhibit lower mechanical strength compared to CNTs/graphene and weaker antimicrobial performance than metal nanoparticles, suggesting opportunities for hybrid systems that combine CDs’ optical advantages with the structural or antimicrobial benefits of other nanomaterials. This positions CD-based films as a versatile, eco-friendly solution for smart packaging where optical sensing and moderate preservation are prioritized over extreme mechanical or antimicrobial performance.

#### 4.1.1. Improvement of Mechanical Properties

Mechanical property evaluation is a key indicator to ensure the reliability of packaging films during food processing, transport and storage. Tensile strength (TS) and elongation at break (EB) serve as the two core evaluation parameters, reflecting the maximum load-bearing capacity and plastic deformation limit of the material before fracture, respectively. These mechanical properties are mainly influenced by the intrinsic properties of the material, preparation process parameters, environmental factors, and microstructural features. CDs can significantly enhance the mechanical properties of polymer matrices by virtue of their unique nanoscale core–shell structure and the abundance of reactive groups on the surface. It improves stress transfer efficiency through interfacial enhancement effects mediated by hydrogen or covalent bonds. It also enables structural densification by suppressing microscopic defects and increasing crystallinity. The triple effects of interface bonding, covalent cross-linking, and structural densification have achieved simultaneous improvements in tensile strength and toughness. The experimental data show that CD composite films exhibit synergistic enhancement in both TS and EB compared to pure polymer films, which fully demonstrates their potential for application as highly efficient nano enhancers in the food packaging field. As shown in Table 4, the TS and EB of the CD composite films showed synergistic enhancement compared to the pure polymer films, confirming their potential as high-performance nano-enhancers in the food packaging field.

It has been shown that the type and amount of CD fillers have a significant modulation effect on the mechanical properties of biopolymer-based composite film. Specifically, the physical properties of composite films doped with CDs are influenced by the surface functional group composition of the CDs, the initial concentration of the film-forming matrix, and the interfacial binding mechanism between the CDs and the matrix. Recent studies reveal that the optimal incorporation of CDs (typically 0.3–5 wt%) can simultaneously enhance mechanical, barrier, and functional properties through nanoscale reinforcement mechanisms. Hong et al. reported that 4 wt% CDs loaded in PVA films increased TS from 35.42 MPa to 41.95 MPa and EB from 273.70% to 381.65% through hydrogen bond-mediated stress transfer [46]. Duan et al. introduced CDs into PVA matrix and successfully prepared packaging films with both high strength and toughness, demonstrating 100 % blocking efficiency for UV, and 99.90 % blocking efficiency for HEBL. The strong hydrogen bonding interactions between the CDs and the PVA matrix suggested that the CDs have the potential to improve the oxygen barrier and thermal stability of PVA films. The TS and EB of the films were remarkably improved with an increasing CD content at 0.4% CDs. This enhancement is mainly attributed to the highly homogeneous distribution of CDs in the film-forming matrix and the strong hydrogen bonding network formed between them, which effectively inhibits phase separation and promotes interfacial stress transfer. However, the mechanical tensile strength and elongation at break of the films decreased when the addition of CDs reached 0.8%, which could be attributed to the agglomeration of CDs [13]. Similarly, Yang et al. investigated the effect of various CD contents on the physicochemical and functional properties of composite films, and found that, compared to 6 wt% based on polymer, the films with 3 wt% of CDs exhibited excellent UV blocking, improved water and gas barriers, and effective antioxidant activity (DPPH and ABTS removal rate over 95%) [49]. CDs have emerged as highly effective polymer reinforcements due to their distinctive nanoscale effects, exceptionally high specific surface area, and abundant surface functional groups. These characteristics enable CDs to significantly enhance the mechanical properties of polymeric materials through both interfacial reinforcement mechanisms and nanoscale strengthening effects. Particularly noteworthy is the superior reinforcing efficiency observed in N, S-co-doped CDs, which clearly demonstrates the advantageous synergistic effects arising from heteroatomic synergy effect. However, exceeding the critical additive level can lead to nanoparticle agglomeration, which in turn leads to stress concentration and mechanical property degradation. Current research in this area still faces several challenges. Firstly, the precise modification and spatial distribution control of functional groups on the surface of CDs are still challenging, especially since the quantitative conformational relationships between functional group types, densities, and material properties have not been fully elucidated. Secondly, maintaining the homogeneous dispersion stability of CDs in the polymer matrix during processing and use represents another challenge. Finally, the safety of CD-based composites in food contact applications needs to be systematically evaluated. Breakthroughs in these key technologies will directly promote the industrialization of CD-based smart packaging materials.

#### 4.1.2. Promoting Barrier Properties

As the core functional elements of food packaging materials, barrier performance through multi-level protection mechanism directly affects the food quality assurance effect [27,50]. A perfect packaging barrier system requires ultraviolet (UV) barrier rate can effectively block the photo-oxidation chain reaction. Water vapor permeability (WVP) and surface hydrophobicity (WCA) are also needed to build a two-way moisture control barrier. In addition, oxygen transmission rate (OTR) and carbon dioxide selectivity (CO_2_/O_2_ transmission ratio) synergistically inhibit aerobic microbial proliferation and lipid oxidation. The synergistic effect of this multiscale barrier network can lead to extended shelf life for different food categories [32]. This section will focus on the core mechanism of barrier properties in food packaging and its influence on food quality patterns.

As a novel nano functional material with UV-protective properties, the utilization of CDs in functional food packaging provides an innovative solution to the preservation of photosensitive food. Firstly, CDs can selectively absorb UV photons in the 280–400 nm band through discrete energy levels generated by the quantum confinement effect, which can effectively inhibit the degradation of photosensitive components [51]. Second, the nano-size properties and surface modification groups of CDs enable molecular-level dispersion in polymer matrices, providing greater than 95% shielding in the UVA band (300–400 nm) while maintaining more than 85% visible light transmittance [52]. In addition, CDs can convert absorbed UV energy into thermal energy to avoid secondary radiation damage, and their biodegradable properties are significantly better than traditional UV absorbers. The efficient protection, visual transparency, and environmentally friendly properties of CDs make CD-doped packaging materials have unique advantages in preserving photosensitive products such as dairy products and high-fat foods, and also provide new ideas for the development of smart active packaging.

The UV barrier function of the CD-doped films was analyzed at 250 nm (UVC), 300 nm (UVB), and 350 nm (UVA). The incorporation of 3% CDs into PVA films demonstrated outstanding UV-blocking performance, achieving shielding rates of 99.9%, 99.9%, and 99.1% against UVC, UVB, and UVA radiation, respectively. Simultaneously, significant enhancements were observed in both mechanical properties and water vapor barrier performance. Furthermore, the PVA film containing 3% CDs displayed 98.9% radical scavenging activity as measured by the ABTS method, along with 100% antibacterial efficacy against both *S. aureus* and *Salmonella enterica* (S. enterica) [53]. The excellent UV-blocking properties are mainly due to the size-dependent bandgap and quantum-limited domain effects of CDs, as well as the abundant functional groups on their surfaces. In addition, the aromatic carbon structure, multiple scattering effects, enhanced interfacial interactions, and surface modification further enhance the UV protection of the films. Notably, the quantum-limited effect of CDs enhances the absorption and scattering of UV light, thereby inhibiting the photochemical degradation of PVA polymers and delaying food spoilage. Tammina et al. also found that N- and P-co-doped CD (NPCD)-modified films exhibited superior UV-blocking properties compared to P-doped CDs (PCDs). The polyethylene glycol (PEG) film loaded with 4% NPCDs demonstrated enhanced UV-blocking performance, increasing from 53.7% to 79.9%. The film also exhibited significant antioxidant activity (DPPH: 12.7%; ABTS: 67%). Furthermore, composite films containing 8% CDs completely eradicated *E. coli* and *Listeria monocytogenes* (*L. monocytogenes*) after 6 h of incubation [54]. This may be attributed to the presence of amino groups on the surface of CDs, which facilitates *n*–π* and π–π* electron leaps and thus enhances UV absorption. This finding provides an important theoretical basis and modification strategy for the design of efficient UV protective materials.

CDs as nano-enhancers can significantly enhance the water vapor barrier properties of packaging films through a multiscale mechanism of action [55]. Water vapor transmission through films involves three successive processes: surface adsorption, analytical diffusion, and interfacial desorption. By precisely regulating the internal water activity of the packaging system, the hydration of microbial cytoplasmic membranes can be effectively limited. The introduction of CDs can achieve this by inhibiting enzyme–substrate molecular interactions and delaying chemical deterioration reactions such as non-enzymatic browning. Firstly, the abundant hydrophilic functional groups on the surface of CDs capture water molecules through hydrogen bonding. In addition, CDs form a dense crosslinked network with polymer chains, which extends the diffusion path of water molecules. Finally, the hydrophilic–hydrophobic equilibrium is optimized by modification strategies such as nitrogen doping, resulting in a lower WVP. This synergistic barrier mechanism can effectively maintain the moisture activity inside the package, inhibit microbial growth and enzymatic reactions, and is particularly suitable for moisture-sensitive products such as bakery products and dry products to preserve freshness.

It was shown that pure gelatin films were hydrophilic and that the WCA values of the films increased with the concentration of CDs. The incorporation of CDs increased the interaction with the film matrix and decreased the effective hydrophilic groups on the film surface [28]. The WVP and water droplet contact angle (WCA) of pure cellulose nanofiber (CNF) films do not change after the addition of CDs to the polymer matrix. After the introduction of CDs, the WCA and WVP decreased slightly, but the decrease was not statistically significant. [48]. However, the addition of nitrogen and phosphorus-doped CDs to chitosan (CS) composite films decreased the WVP values of the films. This may be due to the fact that the density of the films became higher due to the increase in the loading of N, P-CDs, which means that the films became more compact [56]. The WVP values of functional films with N, P-CDs integrated with chitosan and starch binary composites, were not significantly affected [57]. The above studies have shown that the enhancement effect of CDs is governed by the polymer chemistry (e.g., amino groups of CS and hydroxyl groups of CNF). The addition of carbon dots (3 wt%) to chitosan/gelatin films reduced the water droplet contact angle (WCA) of the composite films due to the surface hydrophilic functional groups of the carbon dots. However, the WCA was also not significantly reduced with the addition of 3 wt% CDs. The increase in WVP was not statistically significant in CDs doped composite membranes. This suggests that quantum-sized CDs are unable to block the free space within the polymer matrix and affect the tortuosity of the water molecule pathways [58].

### 4.2. CDs Serve as Active Agents for Regulating the Internal Packaging Environment and Maintaining Food Freshness

**Figure 3:**
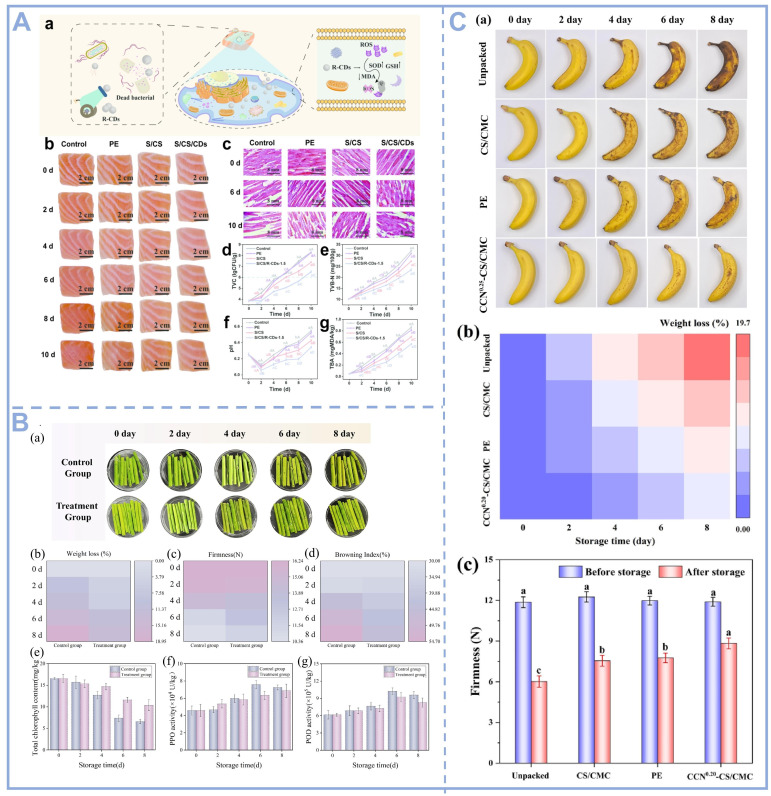
(**A**) Application of R-CDs in salmon fillet preservation: (**a**) antibacterial and antioxidant mechanisms of R-CDs; (**b**) Morphological changes in salmon fillets under different conditions; (**c**) Changes in muscle tissue of salmon fillets under different treatments; (**d**); Changes in salmon fillet TVC; (**e**) Changes in salmon fillet TVB-N; (**f**) Changes in salmon fillet pH; (**g**) Changes in salmon fillet TBA values [59], Copyright (2024) Elsevier. (**B**) Application of lemon-derived CDs in the preservation of blanched asparagus: (**a**) Naked eye images; (**b**) Weight loss; (**c**) Hardness; (**d**) Browning index; (**e**) Total chlorophyll content; (**f**) PPO; and (**g**) POD activity of asparagus during refrigeration [28], Copyright (2025) Elsevier. (**C**) (**a**) Typical images of bananas subjected to various treatments at different times: untreated, PE film, CS/CMC film, and CCN^0.20^-CS/CMC film; (**b**) Weight loss of banana during storage; and (**c**) Change in hardness [63], Copyright (2024) Elsevier.

CDs are a new type of nanomaterials with a smaller size and more excellent biocompatibility, antioxidant, and antimicrobial properties than other carbon-based materials. They could be applied to produce antioxidant films and antimicrobial packaging materials to extend the shelf life of food, and have been tested in diverse fruits, vegetables and meat products such as bananas, trout, asparagus, etc. And, the results showed that they enhanced many critical indicators of food freshness such as total volatile base nitrogen (TVB-N), peroxide value (PV), thiobarbituric acid reactive substances (TBARSs), peroxidase (POD) and polyphenol oxidase (PPO). Wang et al. synthesized nano-composite film coatings by adding R-CDs derived from carrots to starch and chitosan solutions. The inhibition rate of lipid peroxidation by R-CDs was 72.92%, exceeding that of vitamin C (46%). The addition of R-CDs increased the DPPH and ABTS radical scavenging capacities of the films by 84.96% and 98.16%. They tested the coating using salmon fillets as a cured food product (Figure 3A). By calculating the TVC, TVB-N, and TBA of the salmon fillets, it was found that the S/CS/R-CD-coated film extended the shelf life by 4 days [59]. Similarly, glucose- and spermine-derived CDs can be combined with PVA to prepare nanocomposite thin film coatings. The excellent antibacterial activity and antioxidant capacity of the composite film reduced the dripping rate of salmon fillets during storage at 4 °C by 6.66% [60]. In addition, CDs prepared using Bengal rose and riboflavin were also used for the preservation of salmon fillets. The combined action of RR-CDs and ROS reduced biofilm formation, swarming, motility, and extracellular protease production of *Pseudomonas fluorescens* by 48.45%, 25.51%, 52.79%, and 38.67%, extending the shelf life of salmon by 3 days [40]. Experimental data confirm that the nanocomposite packaging system doped with CDs exhibits excellent food preservation efficacy. The material can not only significantly slow down the food spoilage process, but also effectively guarantee food safety.

As an important source of protein in the daily diet, meat has attracted much attention in terms of its nutritional value and safety for consumption. However, high-fat meat products (e.g., fried meatballs, sausages, etc.) are prone to oxidative rancidity and fat oxidation during storage, which not only affects the flavor and texture, but may also produce harmful substances and threaten consumers’ health. Therefore, the development of efficient and safe preservation technologies is of great significance to the meat industry. Researchers used CDs-doped films to package deep-fried meatballs and systematically investigated their preservation effects on high-fat meat products. During 15 d of storage, the addition of only 0.05% CDs significantly reduced the degree of lipid and protein oxidation in fried meatballs [61,62]. The experimental results showed that the CD composite packaging material could effectively inhibit the lipid peroxidation reaction and delay the rancidity process. This study provides a new idea for the preservation of high-fat meat products and highlights the potential application of CDs in food packaging. In addition to meat and its products, the CD composite packaging materials can also be used for the preservation of vegetables and fruits. As shown in Figure 3B,C, Zhang et al. used CDs prepared from lemon to preserve blanched asparagus. The inhibition zone of the film against *S. aureus* was 15.45 mm, and that against *E. coli* was 18.94 mm. In addition, the film retains chlorophyll content, effectively delays browning, and maintains visual appeal [28]. By inhibiting chlorophyll degradation, browning reaction, and endogenous enzyme activity, the visual quality and quality of the samples were effectively maintained. Gong et al. incorporated carbon dots derived from citrus peel into corn starch and carboxymethyl cellulose. The resulting active film demonstrated antibacterial efficiency exceeding 99.9% against *E. coli* and *S. aureus* under visible light, extending the shelf life of bananas by more than four days [63]. In summary, CD-based nanocomposites provide innovative technological solutions for the postharvest preservation of fruits and vegetables and the maintenance of meat quality through multi-target regulatory mechanisms, including but not limited to reactive oxygen species (ROS) homeostatic balance, the inhibition of key enzyme (e.g., PPO, POD, etc.) activities, and microbial growth intervention.

#### 4.2.1. Antioxidant Properties

By virtue of their unique surface functional groups and π-conjugated structural features, CDs exhibit synergistic antioxidant effects by multiple mechanisms [64]. The hydroxyl (•OH), carboxyl (-COOH), and amino (-NH_2_) functional groups enriched on their surfaces can directly scavenge reactive oxygen species (ROS), such as superoxide anion (O^2•−^) and hydroxyl radical (-OH), by supplying electrons or hydrogen atoms [65]. At the same time, their sp2-hybridized carbon nucleus CDs can also absorb the excitation energy of single-linear state oxygen (^1^O_2_) through the π-conjugated system, which leads to its de-excitation to ground state oxygen. More importantly, CDs exhibit enzyme-like catalytic activities that mimic superoxide dismutase (SOD) catalyzing O^2•−^ disproportionation, the catalase-like decomposition of H_2_O_2_, as well as displaying peroxidase-like activity, and this versatile enzyme-like property enables them to undertake the cascade scavenging of a wide range of ROS [66] via hydrogen atom transfer (HAT). Through the synergistic effects of hydrogen atom transfer (HAT), electron transfer (ET), and surface functional group redox, CDs can efficiently neutralize free radicals and convert them into stable molecules, thus significantly enhancing the overall antioxidant efficacy.

**Figure 4:**
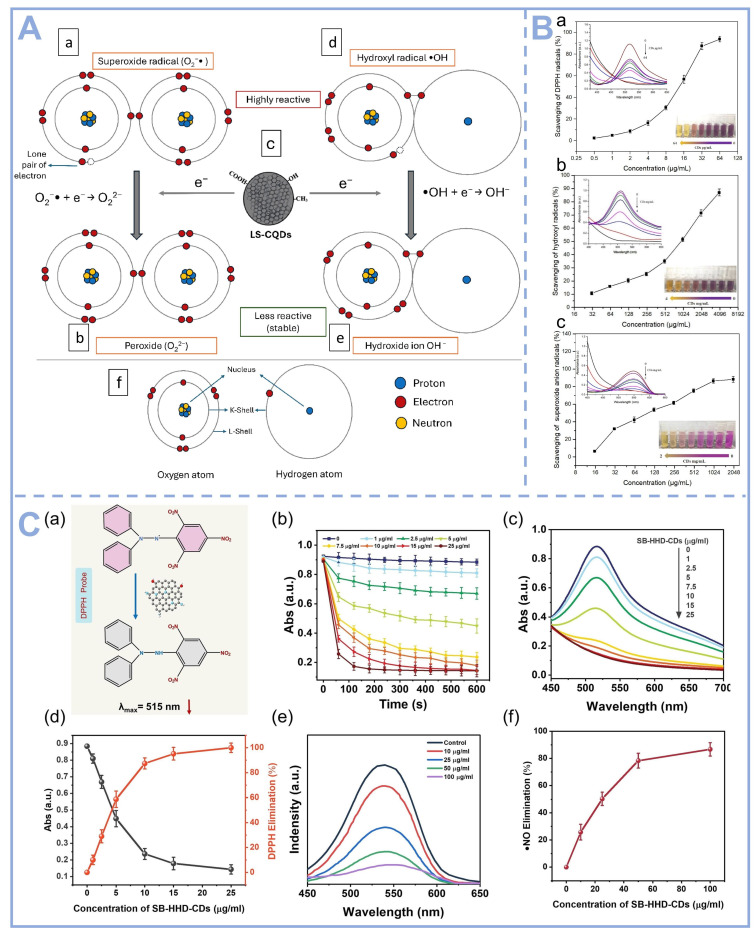
(**A**) Proposed mechanistic pathways for the ROS-scavenging antioxidant activity of LS-CQDs: (**a**) Molecular configuration of the superoxide radical (O_2_^•−^), characterized by an unpaired electron conferring high reactivity; (**b**) Electron transfer from LS-CQDs mediates superoxide radical neutralization, yielding the less reactive peroxide anion (O_2_^2−^); (**c**) Schematic representation of LS-CQDs functioning as an electron-donating moiety; (**d**) Structural depiction of the hydroxyl radical (•OH), a potent oxidative species; (**e**) Hydroxyl radical quenching via electron donation from LS-CQDs, resulting in stable hydroxide ion (OH^−^) formation; and (**f**) Atomic-level illustration of oxygen and hydrogen nuclei with associated subatomic particles [68], Copyright (2025) Elsevier. (**B**) Comparative antioxidant efficacy of CDs against three radical species: (**a**) DPPH• scavenging; (**b**) •OH elimination; and (**c**) O_2_^•−^ neutralization [61], Copyright (2022) Elsevier. (**C**) Kinetic and spectroscopic analysis of SB-HHD-CDs-mediated radical scavenging: (**a**) Proposed reduction mechanism of DPPH by SB-HHD-CDs; (**b**) Reaction kinetics of DPPH reduction at varying SB-HHD-CD concentrations; (**c**) UV–Vis spectral changes of DPPH after 10 min incubation with SB-HHD-CDs; (**d**) Concentration-dependent DPPH elimination efficiency; (**e**) Time-resolved absorption spectra demonstrating •NO radical scavenging by SB-HHD-CDs; and (**f**) Dose–response relationship of SB-HHD-CDs in •NO radical elimination [67], Copyright (2023) Elsevier.

The LS-CQDs synthesized from fresh *L. speciosa* leaves demonstrate potential radical scavenging activity in biological systems, primarily mediated through direct electron donation and H• transfer mechanisms. As depicted in Figure 4A, these carbon quantum dots function as efficient electron donors to neutralize ROS. Specifically, the highly reactive O_2_^•−^ undergoes electron acceptance from LS-CQDs, leading to its reduction into the less reactive O_2_^2−^. Similarly, the •OH, renowned for its extreme oxidative potency, is effectively quenched via electron transfer from LS-CQDs, resulting in stabilization as OH^−^. This dual mechanism highlights the robust antioxidant potential of LS-CQDs in mitigating oxidative stress. CDs prepared by hydrothermal method using banana as a carbon source exhibited significant broad-spectrum radical scavenging ability by Zhao et al. (Figure 4B). The O_2_^·−^ scavenging capacity of CDs increased from 6.32% to 88.21% as their concentration was elevated from 15.625 μg/mL to 2 mg/mL [61]. The scavenging efficiencies of CDs against 1,1-diphenyl-2-picryl-hydrazyl radicals (DPPH), ·OH and O_2_·^−^ were evaluated. The experimental results showed that CDs scavenged a minimum of 88.21% of the above three radicals. This excellent antioxidant performance is mainly attributed to the abundance of active functional groups such as phenolic hydroxyl and carboxyl groups on the surface of CDs, which can efficiently quench free radicals through electron transfer and hydrogen atom transfer mechanisms. As shown in Figure 4C, SBR-HHD-CDs prepared by hydrothermal method using Scutellaria barbata (SB) and Herba Hedyotis diffusae (HHD) as precursors could rapidly scavenge a variety of RONS. SBR-HHD-CDs can eliminate over 80% of the free radicals generated during cigarette smoke combustion [67].

#### 4.2.2. Antimicrobial Properties

CDs exhibit synergistic antimicrobial properties, mainly through the dual pathways of physical damage and chemical interference [69]. In terms of physical effects, CDs can penetrate bacterial cell membranes by virtue of the nano-size effect, and their positive surface charge and negatively charged bacterial membranes can lead to membrane disruption through electrostatic interaction, triggering the leakage of cell contents. In terms of chemical mechanism, CDs can induce ROS outbreak, and their surface functional groups generate a large amount of -OH and O^2•−^ through catalysis, triggering bacterial oxidative stress. Meanwhile, CDs can mimic the activity of antimicrobial enzymes, such as peroxidase, and interfere with the key enzyme system of bacterial metabolism. In addition, the π-conjugated structure of CDs can bind to bacterial DNA/RNA and inhibit nucleic acid replication and protein synthesis. Certain metal-doped CDs (e.g., Ag-CDs) can also release metal ions to enhance the antimicrobial effect [70]. These mechanisms act synergistically so that CDs exhibit broad-spectrum antimicrobial activity against both Gram-positive and negative bacteria, and the bactericidal efficiency can be further enhanced by photodynamic effects under light.

**Figure 5:**
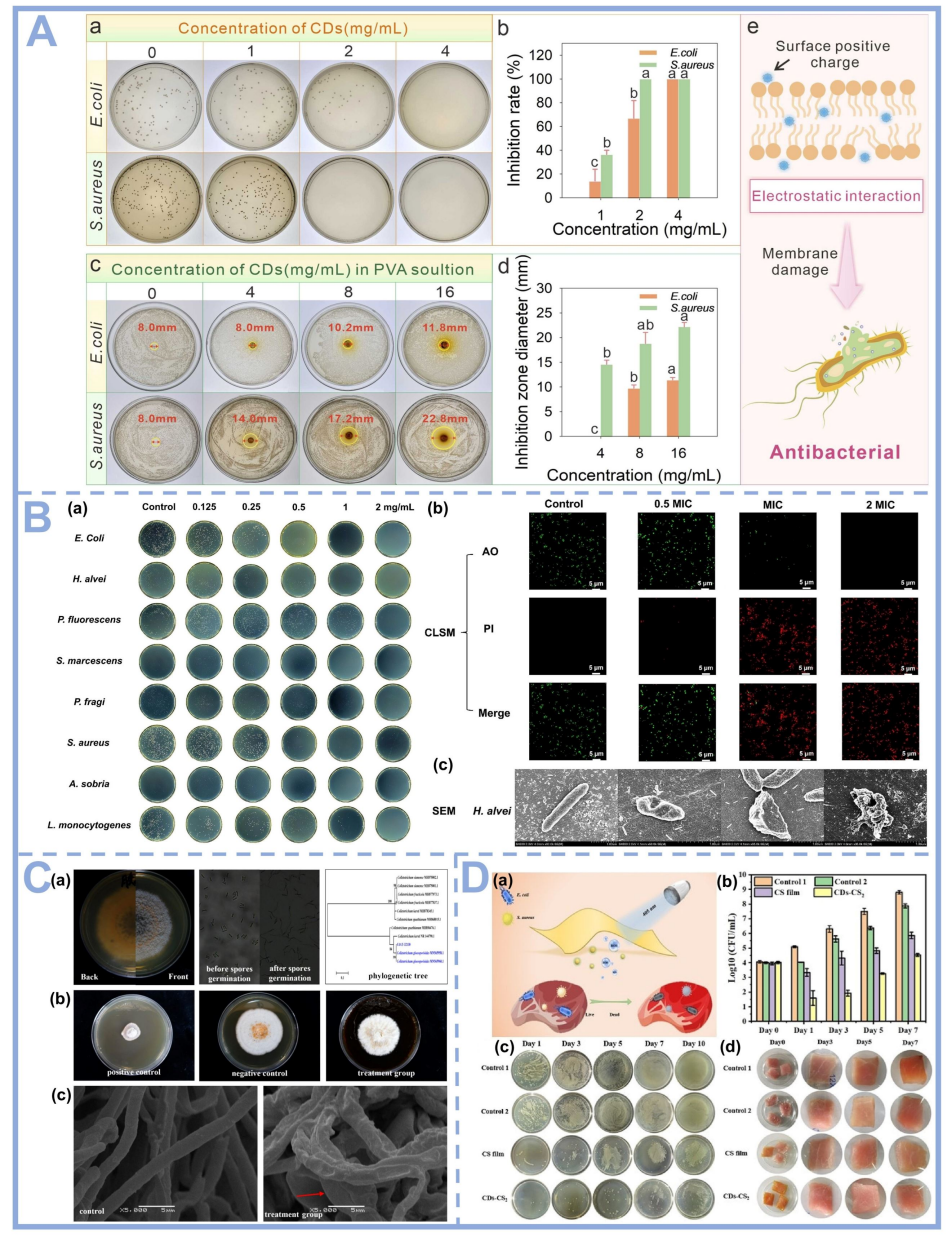
(**A**) (**a**) Bacteriostatic effect at different concentrations of DEF-CDs; (**b**) Antibacterial rate of DEF-CD solution against *E. coli* O157:H7 and S. aureus; (**c**) Zone of inhibition of DEF-CDs/PVA solution against *E. coli* O157:H7 and S. aureus; (**d**) Diameter of zone of inhibition of DEF-CDs/PVA solution against *E. coli* O157:H7 and *S. aureus*; (**e**) Antimicrobial mechanism diagram of DEF-CDs/PVA film [13], Copyright (2025) Elsevier. (**B**) (**a**) Images of inhibition of *E. coli* and various aquatic spoilage bacteria with different concentrations of xβ-CDs; (**b**) CLSM images of *H. alvei* treated with xβ-CDs; (**c**) SEM images of *H. alvei* treated with xβ-CDs [76], Copyright (2025) Elsevier. (**C**) (**a**) Isolation, morphological characterization, and molecular identification of *Colletotrichum gloeosporioides* FJAT-32130; (**b**) Antimicrobial activity of DE-CDs on *C. gloeosporioides* FJAT-32130; (**c**) Effect of DE-CDs on the mycelial morphology of *C. gloeosporioides* FJAT-32130. FJAT-32130 mycelial morphology [77], Copyright (2025) Elsevier. (**D**) (**a**) Antimicrobial strategy of composite films used for pork preservation; (**b**) Total bacterial counts; (**c**) Photographs of bacteria in pork samples packaged at different times; and (**d**) Photographs of pork samples preserved at different times [33], Copyright (2023) Elsevier.

CDs with surface modification and improved functional properties exhibit strong antimicrobial activity against a variety of bacteria, fungi, viruses and other microorganisms. As shown in Figure 5A,B, the CDs exhibited significant antimicrobial activity against *Escherichia coli* (*E. coli*) and *Staphylococcus aureus* (*S. aureus*). As shown in Figure 5A(e), the proton form of amide bonds and amino functional groups on the surface of CDs interact electrostatically with bacterial cell membranes, leading to membrane damage and bacterial death. In addition to bacteria, CDs exhibited inhibitory effects on fungi in Figure 5C, highlighting their great potential for further development as antifungal agents in the field of food packaging materials. On this basis, the surface charge of CDs is an important factor in controlling their bactericidal activity through the adhesion process. Hao et al. successfully synthesized negatively charged NC-CDs and positively charged PC-CDs by controlling the addition of poly (vinyl polyamine) and systematically investigated their antibacterial properties. At the minimum inhibitory concentration dose of PC-CDs, the 4 h bactericidal rates reached 99.21%, 99.92%, 93.68%, and 92.09% against *S. aureus*, *methicillin-resistant S. aureus*, *E. coli,* and *Pseudomonas aeruginosa* [71]. It was found that PC-CDs exhibited strong adsorption to bacteria, whereas NC-CDs were weakly adsorbed to bacterial cells. Zeta potential analysis further confirmed that the positive surface charge of PC-CDs enhanced their anchoring to bacterial membranes, which resulted in a significant enhancement of antibacterial activity. In contrast, NC-CDs showed a weaker inhibitory effect on the tested strains. This result suggests that cationic CDs are able to effectively target bacterial membranes through electrostatic attraction, whereas anionic or neutral CDs have reduced antibacterial efficiency due to charge repulsion.

CDs have attracted considerable attention in studies related to photodynamic therapy (PDT). Under specific wavelength light excitation, the conjugated π structure of the carbon dots absorbs photons to produce electron–hole pairs, and these photogenerated carriers react with the surrounding medium [72]. Firstly, the electrons react directly with dissolved oxygen or water molecules in an incomplete reduction reaction to produce ·O_2_^−^, OH, etc. In addition to this, the excited state energy is transferred to the ground state oxygen (^3^O_2_) to generate ^1^O_2_. These ROS are highly oxidative and irreversibly disrupt the phospholipid bilayer of bacterial cell membranes, oxidize the sulfhydryl groups of key enzyme proteins, and break DNA strands, resulting in efficient bactericidal activity [73,74]. The conjugated structure of carbon dots, doping elements (e.g., N/S), and surface charge (positive charge enhanced membrane binding) can significantly regulate the efficiency and targeting of their ROS generation, and they have the advantages of broad spectral absorption, high biocompatibility, and low drug resistance compared with traditional photosensitizers [75]. As shown in Figure 5D, under illumination with a 405 nm light source, the composite film can generate a large amount of reactive oxygen species. Within 40 min, the film exhibits inhibitory effects on *S. aureus* and *E. coli,* with reduction values of approximately 3.19 and 2.05 Log_10_ CFU/mL. This bactericidal effect was mainly attributed to the continuous production of ROS by carbon dots that inhibited the microbial respiratory chain electron transfer and transmembrane proton gradient, thus blocking key metabolic processes such as ATP synthesis.

### 4.3. CDs as Indicator for Monitoring Food Freshness

**Figure 6:**
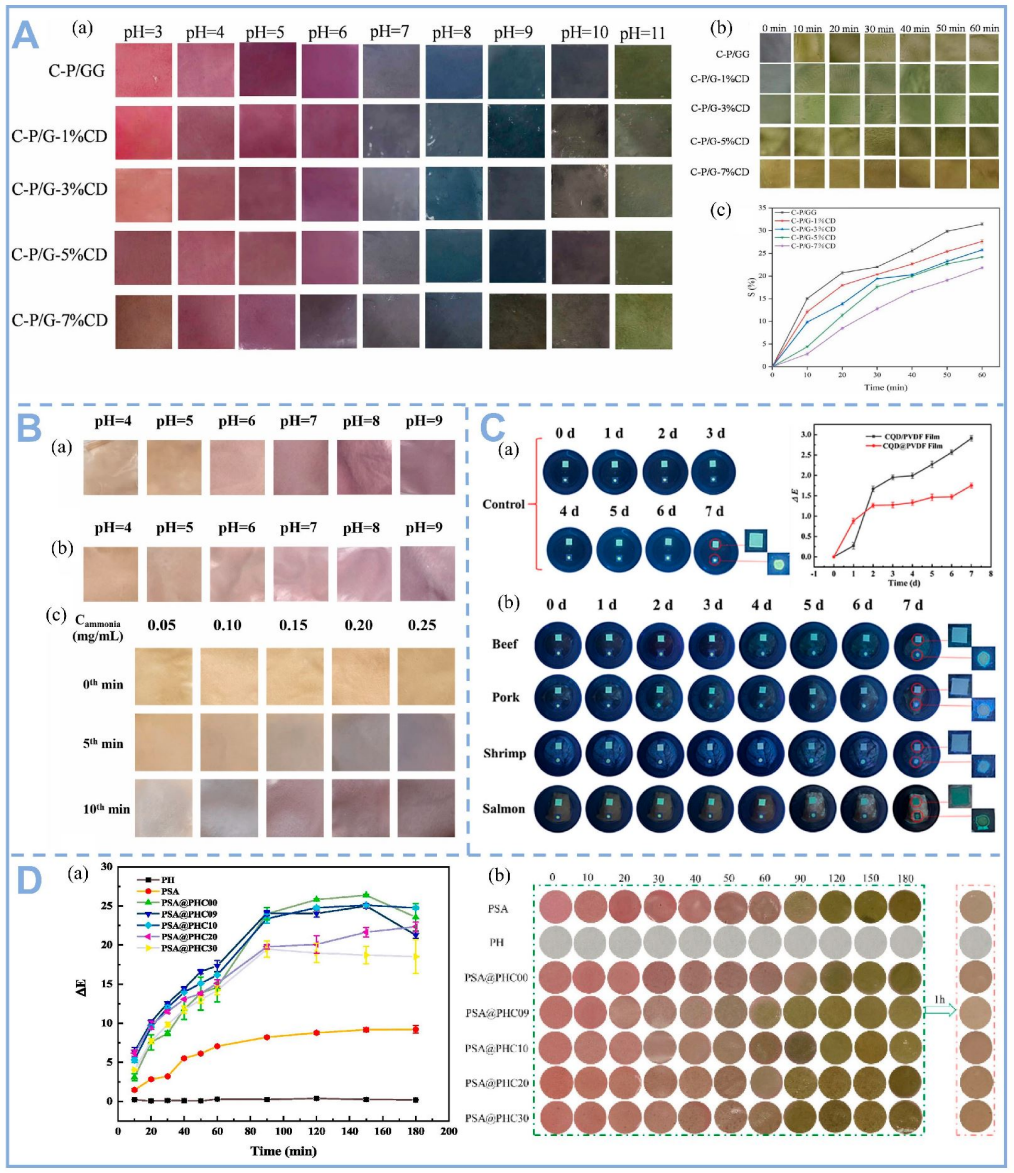
(**A**) (**a**) Color change of different films in buffer solutions at pH 3–11; (**b**) Ammonia exposure and (**c**) relative color difference S value of the film samples [50], Copyright (2024) Elsevier. (**B**) (**a**) Photographs of P-CDs@SP films in different pH solutions before and (**b**) after 30 days; (**c**) Photographs of P-CDs@SP films exposed to different concentrations of ammonia atmosphere for baseline, 5th, and 10th min [80], Copyright (2024) Elsevier. (**C**) (**a**) Photographs of the films in 10 mL of water simulating a highly humid environment during storage (control) and change of the ΔE value of the films at 4 °C; (**b**) Photographs of the films under 365 nm UV light during storage at 4 °C [81], Copyright (2024) Elsevier. (**D**) (**a**) Ammonia-sensitive reactivity and (**b**) discoloration of the film at different times [32], Copyright (2024) Elsevier.

Food freshness is closely related to changes in pH, mainly originating from microbial metabolism and the action of biological enzymes. As food spoils, microbial proliferation breaks down proteins and sugars to produce alkaline volatile amines or acidic metabolites (e.g., lactic acid, acetic acid), which can lead to changes in food pH. Among other things, because of amine accumulation, the pH of meat and seafood is usually elevated upon spoilage. This change can be visually monitored by CDs, and this behavior is mainly attributed to protonation and deprotonation on the surface of CDs [78]. For example, carboxyl-containing carbon dots undergo deprotonation at high pH, triggering a red shift or burst of fluorescence, thus directly reflecting freshness [79]. However, there are limited ways to measure pH using CDs as sensors. As shown in Figure 6A, Liu et al. designed a pH-sensitive colorimetric bilayer film constructed via layer-by-layer self-assembly, using chitosan-loaded purple carrot anthocyanins as the inner indicator layer and gellan gum-incorporated Mg-doped carbon dots as the protective outer layer, which effectively reflects the freshness of pork during storage. As shown in Figure 6B, Ding et al. coated pH-sensitive phosphorus-doped carbon dots (P-CDs) on starch/poly (vinyl alcohol) (SP) films and used them to detect the freshness of pork. It was observed that the color of the packaging film changed from brown-yellow to purple-red when the TVB-N content in pork exceeded the threshold of 15 mg/100 g. This color change can be attributed to the hydroxyl functional groups on the surface of the P-CD/SP composite film, which enhance the hydrophilic properties of the film through stable hydrogen bonding interactions. This in turn accelerated the conversion of TVB-N to ammonia, which ultimately led to an increase in the local pH value [80]. As shown in Figure 6C, CDs show some degree of visible diffusion when using CDs/PVDF films to monitor beef, pork, shrimp, and salmon freshness. As the storage period increases, the color of the film covering beef, pork, and shrimp changes from yellow-green to blue under 365 nm ultraviolet light, while the film covering salmon turns blue-green. Furthermore, when using ratiometric fluorescence for real-time freshness detection, the fluorescence intensity could not be directly discerned with the naked eye [81].

Anthocyanins, as natural pH-responsive pigments, exhibit dynamic structural transformations under varying protonation states, leading to vivid color transitions (e.g., red in acidic to blue/green in alkaline conditions) that correlate with food freshness markers such as TVB-N and biogenic amines. Their rapid, naked-eye-detectable color changes enable real-time qualitative assessment, making them indispensable for visual freshness indicators. Recent advancements have integrated anthocyanins with CDs in bilayer smart labels (Figure 6D), creating a synergistic system where anthocyanins offer immediate visual feedback while CDs enhance functionality through fluorescence-based quantitative detection, achieving ultra-sensitive spoilage monitoring at concentrations as low as 1–10 ppm. The CDs’ outer layer not only safeguards anthocyanins from photodegradation via UV absorption and photostability but also amplifies spoilage signals through photocatalytic TVB-N oxidation, while their inherent antimicrobial properties—mediated by ROS generation and membrane disruption—actively delay microbial growth, thereby extending food shelf life. This innovative dual-mode ‘colorimetric-fluorescence’ output, combined with the dual-function ‘indication-preservation’ mechanism, represents a transformative leap in smart packaging, merging real-time visual monitoring with high-precision fluorescence sensing and active food preservation. Such systems have demonstrated a 30% reduction in spoilage rates in perishable goods like seafood, while IoT-compatible smartphone-based fluorescence/RGB analysis further enables cloud-integrated spoilage prediction, paving the way for next-generation intelligent packaging solutions that enhance food safety, minimize waste, and empower consumers with actionable freshness data.

## 5. Toxicity of CD-Based Films

The biosafety issues that may arise when CD-based packaging films come into contact with food deserve focused attention. In practical food packaging application scenarios, factors such as mechanical action, temperature changes, and long-term use may lead to the release of CDs nanofillers from the films [82]. These released nanomaterials may enter the human digestive system through the food migration pathway and interact with cells after absorption, thus raising potential biocompatibility issues [83]. A study measured the migration of CD composite films in food simulants using 3% acetic acid, and the results showed negligible migration levels [84]. Another study conducted migration tests on nanocomposite films using a 95% ethanol solution as a lipophilic food simulant. The migration amount was only 0.85 mg/dm^2^, less than one-tenth of the international migration limit (10 mg/dm^2^) [85]. Although studies using standardized food simulants (such as 3% acetic acid as specified in the EFSA and OECD protocols) have shown that the migration levels of certain CD composites are negligible, a systematic risk assessment consistent with the international regulatory framework (including the OECD guidelines for the safety testing of nanomaterials and the EFSA requirements for migration analysis and toxicological assessment) remains crucial. For this reason, a systematic assessment of the biotoxicity characteristics of CD materials themselves and their migration patterns in polymer matrices is needed, which is of great significance for ensuring food safety. Based on this, this section will focus on the safety of CD fillers in packaging applications, including potential risk analysis and corresponding risk prevention and control strategies.

Although the cytotoxicity threshold of CDs is significantly higher than that of AgNPs and ZnO, they avoid the risk of chromosome damage associated with AgNPs and ROS-mediated DNA breaks associated with ZnO. Their potential cytotoxicity should not be ignored. Firstly, the cytotoxicity of CDs is affected by precursor materials. Different organic raw materials produce characteristic by-products during high-temperature carbonization, and the presence of these impurities significantly affects the toxicity profile of the end-products. In addition, the surface modification strategy, action concentration, and particle size distribution together constitute a multidimensional system of factors regulating the biological effects of CDs, and the chemical composition of CDs is significantly correlated with their cytotoxicity, which is a core parameter for regulating their biosafety. Studies have shown that CDs synthesized from natural precursors exhibit excellent biocompatibility and low cytotoxicity [86]. The cytotoxicity of CDs also showed a dose-dependent relationship. It was shown that cell survival decreased dramatically from 90% to 80% when the concentration was increased from 1 mg/mL to 10 mg/mL [87]. Another study showed that, even at 20 mg/mL, the cytotoxicity of CDs synthesized by kvass was only insignificant [88]. The analysis results show that there is a synergistic effect between the properties of the precursor material and the action concentration, which jointly determines the toxicity characteristics of CDs. Similarly, the size of CDs plays a crucial role, with smaller CDs exhibiting higher cellular uptake and potentially inducing cytotoxic effects. While it must be acknowledged that CDs generally demonstrate low cytotoxicity, it is noteworthy that specific cytotoxic effects may vary depending on the experimental conditions and cell types [89]. Additionally, several studies have investigated the genotoxicity of CDs, which appears to cause no acute damage to genetic material. A study evaluated the biosafety of photoluminescent CDs synthesized via nitric acid oxidation. This study reported no acute toxicity, genotoxicity, or abnormalities in mouse organs [84].

While CDs exhibit superior biocompatibility compared to conventional nanomaterials, current toxicological assessments present critical limitations that must be addressed—particularly the predominant use of ≤14 d exposure periods in 90% of studies, which fails to capture the 30–180 d safety profiles required for food packaging applications, thereby obscuring the evaluation of chronic low-dose cumulative effects, organ-specific bioaccumulation patterns, and delayed-onset toxicity mechanisms. Furthermore, methodological inconsistencies persist due to the absence of standardized protocols for dose metrics (mass vs. surface area concentration), material characterization in biological matrices, and endpoint selection (cytotoxicity vs. genotoxicity assays), compounded by the variable reporting of parameters influencing toxicity outcomes. These gaps necessitate the establishment of unified testing methodologies through interdisciplinary research encompassing accelerated aging simulations, chronic in vivo toxicological investigations, and environmental fate analyses to comprehensively validate CDs’ safety under real-world usage conditions before widespread commercial adoption.

The cytotoxicity of CDs is critically modulated by surface passivation strategies and physicochemical properties. Functionalization with biocompatible agents (e.g., polyethylene glycol, chitosan, or citric acid) reduces biofilm interactions and cytotoxicity through surface shielding, while chemical modifications like amination or carboxylation enhance stability and mitigate harmful substance release [90]. Concurrently, precise control over particle size and crystallinity minimizes nanoscale membrane disruption and associated toxicological risks. Beyond intrinsic material design, embedding CDs within polymer matrices or constructing core–shell architectures (e.g., SiO_2_-coated CDs) further enhances safety by physically limiting direct food contact and suppressing nanofiller migration.

## 6. Conclusions and Future Perspectives

**Figure 7:**
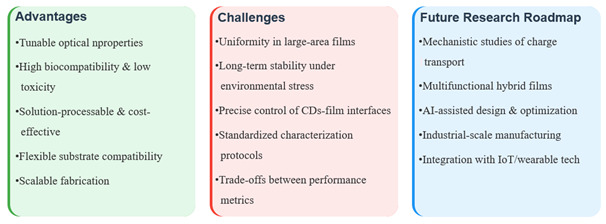
Conceptual diagram summarizing key aspects of CDs-based films for advanced applications.

CDs have emerged as a promising nanomaterial for sustainable food packaging due to their unique optical properties, biocompatibility, and antimicrobial activity. This review investigates the use of biomass-derived CDs for the preparation of biodegradable packaging films, focusing on surface functionalization strategies for the performance enhancement and scalable synthesis routes to achieve cost-effective production. The integration of CDs with polymer matrices significantly enhances mechanical strength, barrier properties, antioxidant/antimicrobial efficacy, and fluorescence-based sensing capabilities, enabling active and smart packaging applications. However, commercialization requires addressing key conversion challenges, including a comprehensive cost–benefit analysis of biomass precursors versus petrochemical alternatives and compatibility with industrial processing methods such as extrusion and coating. Therefore, multifunctional CD films represent a viable next-generation packaging solution awaiting cross-sectoral efforts to standardize manufacturing protocols and safety validation Figure 7.

In conclusion, CDs and their composites show significant potential for application in the field of functional coatings for food packaging. Therefore, there is a need to develop methods for the scale-up preparation of CDs with low-cost and environmentally friendly features to ensure the stability of their properties. To ensure the safe use of carbon dots in various applications, surface modification, and functionalization, the use of natural precursors and the control of carbon dot size through different synthesis and purification methods can be employed during preparation. When preparing composite films, encapsulation and multi-layer barrier films can be used. Furthermore, before widespread adoption can occur, robust interdisciplinary studies must establish harmonized testing methodologies, spanning accelerated aging trials, chronic in vivo toxicology, and environmental fate analyses, to validate safety under real-world usage scenarios. Addressing these knowledge gaps through industry-academia-regulatory collaboration remains essential for transforming CD-based packaging from promising technology to commercially viable, socially respon.

## Figures and Tables

**Table 1:** Comparative analysis of CD synthesis methods for food packaging.

Method	Advantages	Limitations	Food Packaging Suitability	Key Metrics
Top–down	High yield Low material cost	Broad size distribution Toxic by-products (e.g., NOx)	Moderate (requires purification)	Biocompatibility: 70–85% Quantum yield: 10–25%
Bottom–up	Precise size control Excellent biocompatibility	Higher cost Moderate scalability	High (direct food contact)	Migration risk: Low Quantum yield: 20–45%

**Table 2:** Synthesis of CDs from different methods using diverse bio-sourced materials.

Source	Method of Synthesis	Synthetic Conditions	Properties	References
Wheat straw powder	Hydrothermal	180 °C for 12 h	Biodegradability, mechanical properties, and thermal performance	[23]
*M. jalapa* petals	Hydrothermal	200 °C for 6 h	UV-blocking properties (from 96.8 to 99.9%), reduced their water vapor permeability (from 2.91 to 2.13 × 10^−11^ g·m/m^2^·s·Pa)	[24]
Dried mango peel	Hydrothermal	200 °C for 6 h	Enhanced UV-barrier, antioxidant and antibacterial properties of films	[25]
Pineapple leaf	Hydrothermal	200 °C for 12–16 h	Blocked over 95% of UV radiation and exhibited potent antioxidant activity	[26]
*Sargassum fusiforme*	Hydrothermal	175 °C–200 °C for 6 h	Antioxidant activity and antibacterial efficacy against *S. aureus* and *E. coli*	[27]
Lemon peels	Hydrothermal	200 °C for 3 h	UV-blocking capabilities, mechanical strength (38.80 MPa), and antioxidant activity (43.45%)	[28]
Purple hull pistachio	Hydrothermal	190 °C for 8 h	100% UV protection, potent antibacterial activity against *S. aureus* and *E. coli* strains, and antioxidant properties (DPPH; 82.3 ± 0.1% and ABTS; 90.6 ± 0.1%)	[29]
Orange peel	Hydrothermal	160 °C for 6 h	Improved the thermo-oxidative resistance and mechanical properties of the film	[30]
Banana peel	Hydrothermal	200 °C for 2 h	Antioxidant and UV blocking properties	[31]
Highland barley bran	Hydrothermal	200 °C for 10 h	Generate ROS and impede bacterial proliferation	[32]
Turmeric	Hydrothermal	180 °C for 12 h	exhibited reductions of approximately 3.19 and 2.05 Log_10_ CFU/mL for *S. aureus* and *E. coli*	[33]
Elephant grass leaves	Hydrothermal	1.05 bar, 121 °C for 40 min	Inactivate 69.9 ± 1.7, 76.4 ± 1.8, and 99.92 ± 0.03% of attached *E. coli* cells	[34]
Enoki mushrooms	Hydrothermal	200 °C for 6 h	Antioxidant activity in the DPPH and ABTS	[35]

**Table 3:** Comparative analysis of CDs versus other nanofillers in composite film applications.

Nanofiller Type	Advantages	Disadvantages	Key Differentiators of CDs
CDs	Excellent biocompatibility Tunable fluorescence Low toxicity Water solubility Easy functionalization	Lower conductivity than carbon nanotubes Limited mechanical reinforcement Batch-to-batch variability	—
Metal Nanoparticles	High electrical/thermal conductivity Plasmonic effects Antimicrobial properties	Potential cytotoxicity High cost Oxidation issues	CDs are non-toxic and cheaper
Carbon Nanotubes	Exceptional mechanical strength High conductivity Thermal stability	Poor dispersion Potential pulmonary toxicity Complex functionalization	CDs disperse easily and are biocompatible
Graphene	Ultra-high surface area Excellent conductivity Mechanical strength	Restacking issues Expensive production Limited luminescence	CDs offer intrinsic fluorescence
Cellulose Nanocrystals	Biodegradable Low cost High stiffness	Hydrophilic (moisture sensitive) No electrical functionality Limited optical properties	CDs provide optoelectronic functionality

**Table 4:** Mechanical properties and barrier performance of CD-based packaging films.

Source	Substrate	Proportion	Mechanical Properties	Barrier Performance	Food Types	Ref.
*Sargassum fusiforme*	*Sargassum fusiforme* Polysaccharides/Gelatin	1 wt %	TS: 18.02 MPa→32 MPa EB: 46.87%→100%	OTR: ↓0.85 × 10^−3^ g/m^2^ s Pa →	Blueberry	[27]
Sodium citrate, ethyl ferulate, ethylenediamine	Polyvinyl alcohol	0.8 wt%	/	UV–vis-shielding: 0.59→0.06 (OD_445_)	Strawberries, jujubes, and pasteurized milk	[13]
Glucose	Chitosan/gelatin	2 wt% based on polymer	TS: 79.5 MPa→82.3 MPa EB: 7.3%→8.2%	T_280_: 4.5%→0.03%, T_600_: 90.9%→82.5%	Avocado	[42]
Potato skins	Gelatin	4 wt% based on polymer	TS: 60.7 MPa→50.3 MPa EB: 12.2%→11.8%	WVP (×10^−10^ g·m/m^2^·Pa·s): 7.9% →9.9%, WCA: 70.7°→61.5 °	/	[43]
Coffee ground	Chitosan	5 wt% based on polymer	TS: 40.15 MPa→48.24 MPa EB: 4.39%→4.01%	WCA: 71°→101°, UV-C-shielding: 21.49→99.17, UV-B-shielding: 19.36→96.58, UV-A-shielding: 18.86→93.76,	Shrimp	[44]
Chitosan	Fish gelatin	5 wt% based on polymer	TS: 23.5 MPa→36.2 MPa EB: 47.1%→46.8%	WVP (×10^−10^ g·m/m^2^·Pa·s): 9.47%→9.05%	Pacific white shrimps	[45]
Citric acid and urea	PVA/glycerol	0.16 wt%	TS: 35.42 MPa→41.95 MPa EB: 273.7%→381.65%	WVP (×10^−9^ g·m/m^2^·Pa·s): 8.11%→6.76%, WCA (deg.): 49.18→67.1	Shrimp	[46]
Jute	PVA	2.5 wt% based on polymer	TS: 1 MPa→1.05 MPa EB: 111.7% →134.93%	WVTR (gm^−2^ h^−1^): 118.89 →115.49	Banana, plum	[47]
Coconut husk	PVA	2.5 wt% based on polymer	TS: 1 MPa→1.25 MPa EB: 111.7%→181.2%	WVTR (gm^−2^ h^−1^): 118.89 →101.91	Banana, plum	[47]
Banana	PVA	2.5 wt% based on polymer	TS: 1 MPa→1.50 MPa EB: 111.7%→181.2%	WVTR (gm^−2^ h^−1^): 118.89→98.51	Banana, plum	[47]
Water hyacinth	PVA	2.5 wt% based on polymer	TS: 1 MPa→1.72 MPa EB: 111.7%→223.8%	WVTR (gm^−2^ h^−1^): 118.89 →95.11	Banana, plum	[47]
Pullulan, ethylenediamine, citric acid	Gelatin/ CNF	5 wt% based on polymer	TS: 53.0 MPa→15.0 MPa EB: 9.1%→17.5%	T_280_: 40.2%→2.1%, T_600_: 89.2%→87.6%	Egg	[48]
